# Preliminary Development and Assessment of a Multiplex Assay for Human Papillomavirus Antibody Detection Using Commercial L1 Proteins

**DOI:** 10.1002/jcla.70343

**Published:** 2026-09-01

**Authors:** Arash Aslanabadi, Maryam Karimi, Laura Powell, Lisa Schumaker, Ashley Shutt, Mahsa Hojabri, Rahim Abbasi, Søren M. Bentzen, Kevin J. Cullen, Trevor A. Crowell, Rebecca G. Nowak, Mohammad M. Sajadi

**Affiliations:** ^1^ Institute of Human Virology University of Maryland School of Medicine Baltimore Maryland USA; ^2^ University of Maryland Greenebaum Comprehensive Cancer Center University of Maryland School of Medicine Baltimore Maryland USA; ^3^ U.S. Military HIV Research Program, CIDR Walter Reed Army Institute of Research Silver Spring Maryland USA; ^4^ Henry M. Jackson Foundation for the Advancement of Military Medicine Bethesda Maryland USA

**Keywords:** antibody, diagnosis, HPV, infection, multiplex, vaccine

## Abstract

**Background:**

Human papillomavirus (HPV)‐associated cancers are preventable with vaccination. Serologic assays monitor antibody responses, but their complexity and cost pose limitations when evaluating population level immunity. We developed an L1‐based assay for the detection of multiple type‐specific HPV antibodies.

**Methods:**

The Luminex‐based assay used recombinant L1 proteins from seven HPVs (HPV‐6/11/16/18/35/45/58) for the detection of type‐specific immunoglobulin (Ig)G and IgA. Cutoffs were established using plasma from infants and children. The assay was optimized and qualified using a sample set from a blinded proficiency panel developed at the Frederick National Laboratory for Cancer Research.

**Results:**

Sensitivity and specificity for anti‐HPV‐6/11/16/18/58 IgG were 100% at the Mean + 3 standard deviation [SD] and Mean + 5 SD thresholds. Sensitivity for HPV‐45 was 100% and 92.9% at the +3 SD and +5 SD thresholds, respectively. IgA detection showed moderate positivity among vaccinated individuals, with peak estimates of 96.4% (HPV‐11) and 85.7% (HPV‐58) at the +3 SD threshold. IgA was correlated with IgG (Spearman's *ρ* = 0.74). The L1‐assay differentiated high, intermediate, low, and negative response groups from the blinded samples for all HPV genotypes (*p* < 0.0001).

**Conclusion:**

The L1‐binding assay has the potential to be a scalable option for high‐throughput detection of broad‐spectrum HPV immunoglobulins, but further standardization and validation are needed.

## Graphical abstract

This study developed and assessed a new multiplex assay for the simultaneous detection of antibodies against multiple Human Papillomavirus (HPV) types. Using recombinant L1 protein, the assay quantifies IgG and IgA responses in human plasma, enabling comprehensive immune profiling across multiple high‐risk cancer‐associated HPV genotypes. This approach provides a high‐throughput, sensitive, and specific platform for HPV seroepidemiological and vaccine studies.

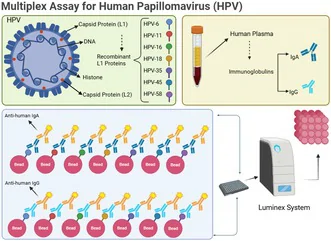

## 1 Introduction

With HPV being the most prevalent sexually transmitted viral infection globally, most sexually active men and women will contract at least one genital HPV infection in their lifetime [1]. HPV types 16 and 18 cause approximately 70% of cervical cancer cases worldwide [2, 3]. Despite the success of prophylactic vaccines [4], HPV remains a significant public health issue, especially in regions with lower vaccination rates and high incidences of cancers [5, 6]. Given oncogenic HPV's strong association with malignancies such as cervical, vulvar, anal, and oropharyngeal cancers [7, 8], accurately assessing population levels of immunity may help streamline vaccination programs when challenged by resource limitations.

Serologic binding assays provide valuable insight into broad spectrum antibodies, both functional and non‐functional, that indicate past infections and vaccine‐induced immunity [9]. Natural infections because of the virus's epithelial confinement may elicit a weak or undetectable antibody response, while HPV vaccination reliably induces a strong systemic antibody response [10, 11]. Therefore, serologic assays sensitive enough to broadly capture a range of antibody responses can help inform vaccine implementation programs on population levels of immunity.

There are several serologic binding methods that vary in their design and types of immune responses captured. Polyclonal binding assays measure broad‐spectrum antibodies that are both neutralizing and non‐neutralizing for multiple HPV type‐specific antigens [12]. These assays originated as enzyme‐linked immunosorbent assay (ELISA) that used HPV type‐specific capsid proteins assembled into virus‐like particles (VLPs) which mimic the conformational structure of the native virus and are the antigen in the vaccines [13, 14, 15, 16, 17, 18]. The serologic binding assays evolved from detecting a single HPV genotype to multiple HPV genotypes [19, 20, 21, 22, 23, 24]. While these VLP‐based assays are highly specific, they require the expression and assembly of multiple recombinant viral proteins in specialized cell lines that can be technically demanding, time‐consuming, and expensive to construct [25]. The other polyclonal binding assay is the GST (glutathione S‐transferase)‐fusion protein assay that, unlike the VLP‐based assays, uses the L1 capsid protein linked to GST [26]. However, this assay has lower sensitivity and accuracy than VLP based assays [25, 27, 28, 29] and may be less optimal for capturing population immunity.

In this study, we present a polyclonal binding assay that uses recombinant L1 proteins to detect broad‐spectrum antibodies to multiple HPV genotypes. This approach bypasses the need for labor‐intensive VLP production while maintaining the antigenicity of the L1 protein. We evaluated our serologic assay's ability to detect HPV type‐specific IgGs and explored its ability to detect HPV type‐specific IgAs. To evaluate basic performance of our assay, we evaluated its sensitivity and specificity using a limited set of samples included in the proficiency panel developed at the HPV Serology Laboratory at the Frederick National Laboratory for Cancer Research.

## 2 Methods and Materials

### 2.1 Antigens and Microsphere Beads

Recombinant L1 proteins from seven HPV genotypes (HPV‐6/11/16/18/35/45/58) were used as antigens for the serologic assay. The choice of antigens was based on the most prevalent anal HPV genotypes in Nigeria [30]. These proteins were covalently coupled with magnetic microspheres for use in the Luminex multiplex system. The coupling efficiency was optimized with 5 μg of antigen per bead set, corresponding to regions no. 12, 14, 19, 22, 27, 34, and 42 of internally labeled carboxylated microspheres.

The following proteins were used:

Antigens: Recombinant L1 proteins for HPV‐6 and HPV‐11 were purchased from Creative Diagnostics (Shirley, NY, Catalog #DAG‐P2510 and #DAG‐P2518, respectively). HPV‐16 and HPV‐18 L1 proteins were obtained from MyBioSource (San Diego, CA, Catalog #MBS318636 and #MBS318652, respectively). HPV‐35, HPV‐45, and HPV‐58 L1 proteins were sourced from Creative Biomart (Shirley, NY, Catalog #HPV35‐004H, #HPV45‐005H, and #HPV58‐002H).

Monoclonal Antibodies (mAbs): Monoclonal antibodies for HPV‐6, HPV‐11, HPV‐16, and HPV‐35 were obtained from Creative Diagnostics (Catalog # CABT‐B880339, CABT‐B8804, CABT‐CS176, and CABT‐CS276, respectively). The antibody for HPV‐18 was purchased from Abbexa (Cambridge, UK, Catalog # abx110595), the antibody for HPV‐45 from Alpha Diagnostic (San Antonio, TX, Catalog #HPV45L41‐S), and the antibody for HPV‐58 from Creative Diagnostics (Catalog #CABT‐B8811).

### 2.2 Controls

#### 2.2.1 Positive Controls for IgG Assay

Monoclonal antibodies (mAbs) against the L1 proteins of the respective HPV serotypes were used as positive controls, sourced as detailed above.

#### 2.2.2 Negative Controls for All Assays

Negative controls consisted of plasma samples from 10 infants who were between 1 and 2 years of age, and plasma samples from 16 children aged 10–12 years (Boca Biolistics, Pompano Beach, FL). Older children were included as they are closer in age and immunologic status to adults, to better reflect the breadth of background antibody responses (and possible cross‐reactivity) that could occur with exposure to different pathogens. Infant ages were chosen to preclude any remnant circulating maternal antibodies, and children's ages were chosen for low likelihood of vaccination, depending on geographic location and vaccination guidelines at the time of sampling. Both groups were presumed to be at low risk for HPV infection.

### 2.3 Coupling of Target Antigens to Beads

The seven recombinant L1 proteins from HPV type‐specific genotypes were conjugated to spectrally unique magnetic carboxylated microspheres using the Luminex coupling kit (Austin, TX, Catalog No: 40‐50016). Each antigen was covalently coupled to a different bead set, corresponding to magnetic microspheres. The conjugation was performed based on the manufacturer's instructions, with some modifications detailed below.

Before the conjugation process, 1–5 million microspheres were prepared by sonicating and vortexing. They were then placed on a magnetic rack, allowing the magnetic microspheres to adhere to the magnets. The beads were washed three times with activation buffer (0.1 M NaH_2_PO_4_, pH 6.2), resuspended, and prepared for activation.

For activation of the carboxyl groups on the bead surfaces and stabilization of the transient intermediate formation, 1‐ethyl‐3‐(3‐dimethylaminopropyl) carbodiimide hydrochloride (EDC; ThermoScientific Pierce, Waltham, MA) and sulfo‐N‐hydroxysulfosuccinimide (sNHS; Thermo Scientific Pierce) were added to the bead suspension. The mixture was incubated on a tube mixer to promote efficient coupling.

Based on the manufacturer's recommendation, 5 μg of antigen per 1 million beads was used. Bead‐protein conjugation was performed by incubating the microspheres with the corresponding proteins for 2 h in the dark at room temperature on a tube rotator set at 25 rpm. After the incubation, unbound antigens were removed with Wash Buffer (Luminex, Catalog # 11‐25167), and active sites on the beads that were not coupled to antigens were blocked.

The concentration of the microspheres was measured using a LUNA‐FL cell counter, and the beads were stored at 4°C in the dark until further use.

### 2.4 Confirmation of Conjugation

To evaluate the efficiency of the antigen coupling, a confirmation assay was performed using serial dilutions of monoclonal antibodies or positive sample controls specific to each protein. These controls were also used to optimize the antigen coupling concentration for each bead region. Once optimized, both positive and negative samples were applied to set up screening conditions and assess the reactivity of the antigen‐coated beads. The entire procedure followed the Luminex guidelines, as per the manufacturer's instructions.

### 2.5 Multiplex Assay

The Multiplex HPV assay was run as detailed below. Following bead preparation, 25 μL of the 10 μg/mL mAb or 1:100 diluted plasma was added to each well of a 96‐well plate containing 50 μL of the bead suspension. All samples were tested in duplicate. While protecting from direct light, the plate was incubated for 1 h at room temperature (RT) with gentle shaking (300–500 rpm), allowing the antibodies to bind to the antigen‐coupled beads. This shaking ensured proper mixing and optimal antibody binding. After the incubation, the plate was washed three times with 200 μL of PBS‐TBN using a magnetic plate washer to remove unbound antibodies and plasma components. In each wash step, 200 μL of PBS‐TBN was added, followed by 2–3 min of incubation before the buffer was aspirated. The magnetic plate washer holds the antigen‐bound beads securely while washing away unbound material, maintaining the integrity of the assay. After washing, 50 μL of phycoerythrin (PE)‐conjugated anti‐human IgG (Millipore, Burlington, MA, Catalog No: SIG‐HC19‐PEIGG) was added to each well. The plate was then incubated for 30 min at RT with gentle shaking. Following the secondary antibody incubation, the plate was washed again three times and was read using the Luminex MagPix system, with the instrument set to measure 50 μL per well. The system ensures that at least 100 beads from each region are analyzed, yielding robust data. The PE‐conjugated secondary antibody provides the fluorescence signal, which is detected and measured by the Luminex system. Results were generated using Luminex xPONENT software, which reports the results as median fluorescence intensity (MFI) values for each antigen‐specific bead set.

Each assay plate included two wells containing only dilution buffer to serve as a blank signal. Additionally, corresponding monoclonal antibodies (mAbs) as positive controls and one constant negative control were included in every assay run to evaluate the performance of the test and ensure consistency across plates.

To control for minor variations between assay plates, all MFIs were adjusted according to the negative control signal from the cut‐off determination run. Specifically, the percentage difference between the negative control from the cut‐off run and the test run was applied to all samples in the test run.

### 2.6 Proficiency Panel Testing

To further evaluate the performance of our assay, we tested a sample set from a blinded proficiency panel developed by Miller et al. [31]. Although this panel was designed to evaluate assay performance for established and validated assays, here we utilized these samples as a source of known positives at various levels to evaluate in a preliminary assessment the ability of our assay to accurately measure negative, low, intermediate, and high levels of HPV type‐specific IgG antibodies. The sample set included 28 HPV vaccinees and 12 negative controls.

### 2.7 IgA Serology

For IgA serology, we followed the same protocol outlined for IgG detection, with minor adjustments. A phycoerythrin (PE)‐conjugated anti‐human IgA (Millipore, SIG‐HC19‐PEIGA) was used as the secondary antibody for detecting IgA responses. Due to the absence of established secondary monoclonal antibodies (mAbs) against HPV type‐specific genotypes for IgA detection, we used plasma that we found to have high IgG reactivity and correspondingly high IgA reactivity (> 10× negative control) as our positive control. The negative control samples were identical to those used for IgG assays, consisting of plasma from infants and children.

### 2.8 Statistical Analysis and Cut‐Off Determination

To establish the seropositivity threshold for each HPV type, cutoff values were determined by analyzing the Net‐MFI (Median Fluorescence Intensity) values from the entire group of 26 infants and children. The mean and standard deviation (SD) of the Net‐MFI values were calculated for each HPV type. To account for outliers, we planned to exclude any Net‐MFI value exceeding the Mean + 5 SDs for the IgA and IgG negative panels, with this process repeated iteratively until no further outliers were detected. Additionally, a negative control sample was included on every run. Net‐MFI values for all samples were normalized based on the observed minor differences in the negative control sample and the cutoff value, ensuring consistency and comparability of results.

A Mann–Whitney test was used to test differences between infants and children's plasma. Kruskal–Wallis tests were used to compare median MFIs by antibody response groups in the proficiency panel. IgG and IgA antibody MFIs were log‐transformed (log_10_) and visualized for correlation using a scatter plot. The IgG and IgA correlation was calculated using Spearman's rank correlation (*ρ*). For all analyses, a 2‐sided type I error of 5% was set for statistical significance. All data analyses were performed using GraphPad Prism (v9.2.0, GraphPad Software, Boston, MA) and Stata 18.0 (StataCorp LLC, College Station, TX).

### 2.9 Ethics Statement

The samples were obtained through different sources. Plasma samples from infants (1–2 years of age) were obtained from previously established cohorts conducted by our research group, in which informed consent was obtained through the parents (UMB IRB approval number HP‐00094782). Plasma samples from children (10–12 years of age) were purchased from Boca Biolistics (Pompano Beach, FL, USA). Samples from the HPV serology proficiency panel were provided by the HPV Serology Laboratory at the Frederick National Laboratory for Cancer Research and were received in a blinded and de‐identified format. The study was conducted in accordance with institutional and ethical guidelines for research involving human specimens.

## 3 Results

### 3.1 Cut‐Off Determination

**FIGURE 1:**
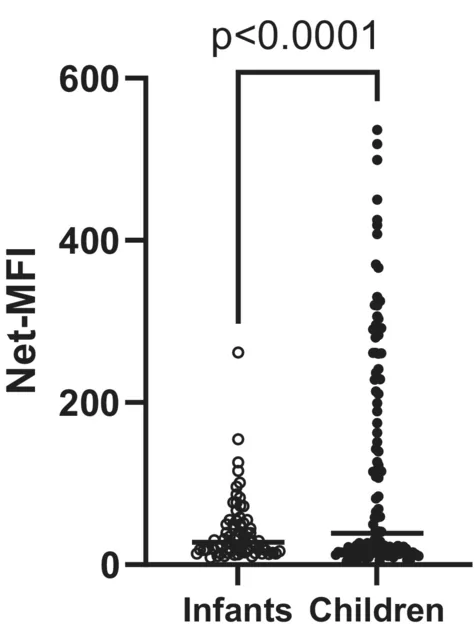
Comparison of net median fluorescence intensity (Net‐MFI) values between infants and children for all HPV genotypes. Each dot represents an individual sample, with horizontal lines indicating the median values. A significant difference in Net‐MFI was observed between infants and children (*p* < 0.0001).

When determining the reactivity of plasma in the negative control group, we noted a significant difference in the Net‐MFI values between infants and children, with the children aged 10–12 having higher values than the infants after pooling across all HPV genotypes (*p* < 0.0001) (Figure 1). Including older children allowed for better calibration of the seropositivity threshold by reducing the likelihood of false‐positive results in subsequent adult analyses.

### 3.2 Sensitivity and Specificity

After setting the cut‐off value for each antigen, we ran a 40‐sample set from a blinded proficiency panel on the L1‐binding assay. Across all samples in the proficiency panel, the assay achieved 100% sensitivity for HPV‐6, 11, 16, 18, 45, and 58 at the +3 SD threshold. At +5 SD, sensitivity remained 100% for all types *except* for HPV‐45 (which dropped to 92.9%).

Because the proficiency panel had not been validated for IgA, we could not calculate the sensitivity. However, we applied the same algorithm for plasma IgG Net‐MFI signals to plasma IgA. Based on this, the percentage positive for HPV‐6, 11, 16, 18, 45, and 58 was calculated as 64.3%, 96.4%, 75%, 67.9%, 57.1%, and 85.7%, respectively, at the Mean + 3 SD threshold. At the Mean + 5 SD threshold, the percentage positive for plasma IgA detection for HPV‐6, 11, 16, 18, 45, and 58 was calculated as 53.6%, 96.4%, 57.1%, 46.4%, 57.1%, and 60.7%, respectively. The percent positive for HPV‐35 IgG and IgA was 50% and 14.3% at the Mean + 3 SD threshold and 35.7% and 14.3% at Mean + 5 SD, respectively.

Specificity with this limited set of samples was also high, with the assay correctly identifying all negative samples. At Mean + 5 SD threshold, specificity for HPV‐6, 11, 16, 18, 35, 45, and 58 was calculated as 100% and remained at 100% at Mean + 3 SD for both plasma IgG and IgA.

### 3.3 High Antibody Response Group

**FIGURE 2:**
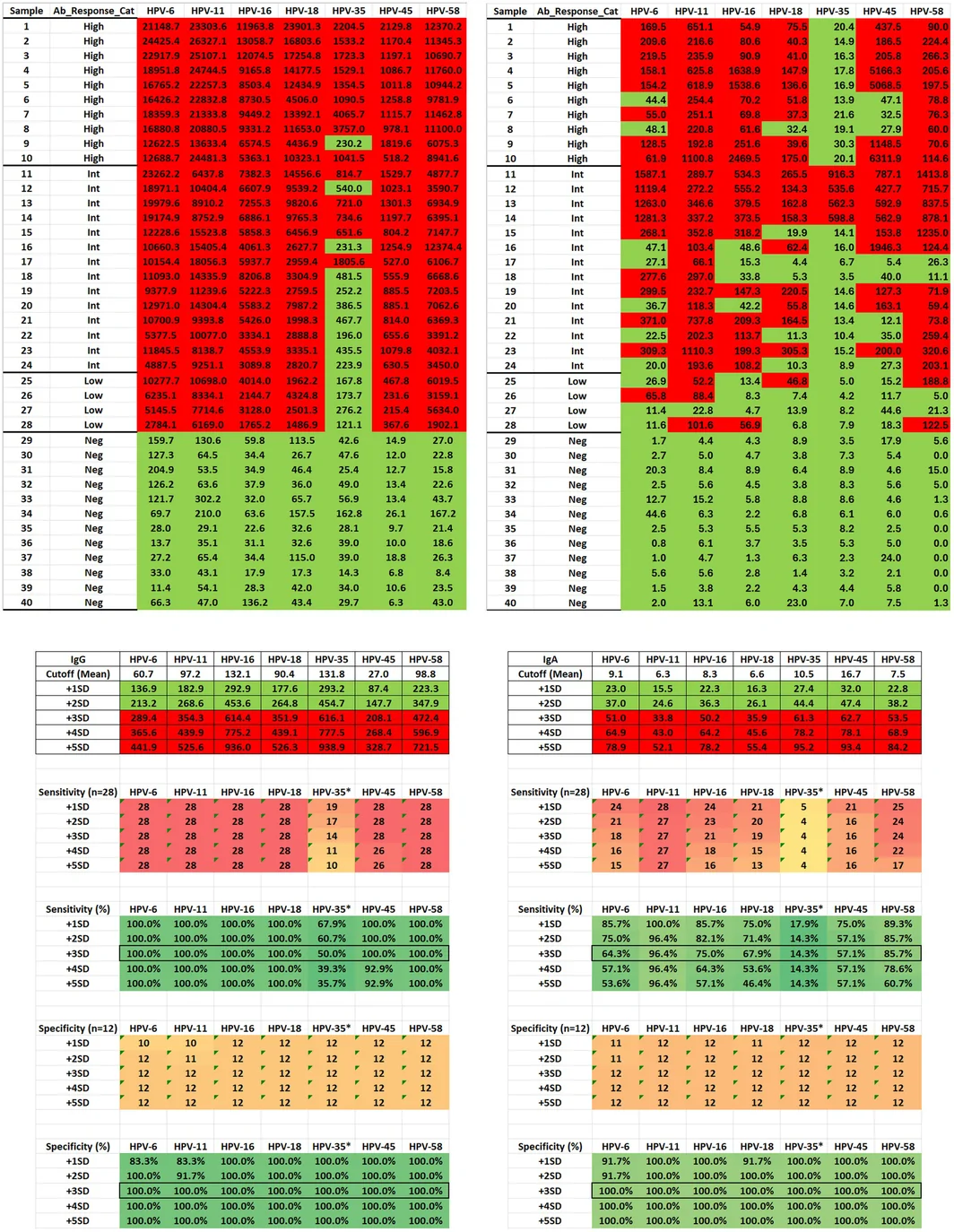
Serologic responses were measured for IgG (left) and IgA (right) in a sample set of proficiency panel samples revealed later as high, intermediate (Int), low, or negative (Neg) responders. Top Panel: Heatmaps display individual MFI (median fluorescence intensity) values for each HPV genotype, with positive responses represented in red and negative responses in green. Bottom panels include cutoff values based on the mean and standard deviations (SD) and sensitivity and specificity calculations at various thresholds. Sensitivity and specificity percentages are presented for different SD cutoffs, highlighting assay performance in distinguishing seropositive and seronegative samples. Note that the antibody response categories (high, intermediate, low and negative) of the sample set are only relevant for IgG. The 4 response categories were not standardized for IgA and were only included as a comparison.

From the proficiency panel, 10 samples had been predefined by the HPV Serology Laboratory at the Frederick National Laboratory for Cancer Research as having high IgG antibody responses. These individuals had all received the 9‐valent HPV vaccine, which included L1 antigens from HPV‐6, 11, 16, 18, 45, and 58. In these 10 samples, IgG reactivity was detected across the included HPV types. Net fluorescence intensity (Net‐MFI) values for HPV‐6 ranged from 12,623 to 24,425 (Figure 2, top left panel). For HPV‐11, Net‐MFI values varied from 13,633 to 26,327, while Net‐MFI for HPV‐16 ranged from 5363 to 13,059. Similarly, HPV‐18 showed Net‐MFI values between 4437 and 23,901. The remaining types—HPV‐45 and HPV‐58—exhibited Net‐MFI values ranging from 518 to 2130 and from 6075 to 12,370, respectively.

### 3.4 Intermediate Antibody Response Group

Fourteen samples were predefined as having intermediate IgG antibody responses. These individuals had detectable immune responses but at a reduced magnitude compared to the high IgG antibody response group. IgG MFI values in this group showed greater variability, with HPV‐6 and HPV‐11 yielding values ranging from 4887 to 23,262, and HPV‐16 showing values from 3090 to 8207 (Figure 2, top left panel). For HPV‐18, the IgG MFI values ranged from 1998 to 14,557. These results suggest that while IgG antibody reactivity was lower, individuals in this group retained detectable immune responses.

### 3.5 Low Antibody Response Group

Four samples were classified as having low IgG antibody responses, potentially due to waning immunity post‐vaccination or another cause. In this group, IgG MFI values for HPV‐6 ranged from 2784 to 10,278, while values for HPV‐16 and HPV‐18 were considerably lower, ranging from 1765 to 4014 for HPV‐16 and 1487 to 4325 for HPV‐18 (Figure 2, top left panel).

### 3.6 HPV‐Negative Group

The assay's specificity was assessed using 12 samples from the proficiency panel that were predefined as negative for HPV‐specific antibodies. These individuals had no known history of vaccination. MFI values for IgG in this group were consistently low and below the positivity threshold across all HPV genotypes, with values ranging from 11.4 to 204.9 for HPV‐6 and 29.1 to 302.2 for HPV‐11 (Figure 2, top left panel). These low values confirmed the assay's ability to differentiate between HPV‐vaccinated and non‐vaccinated individuals, with no false positives recorded among the HPV‐negative group.

### 3.7 Distribution of MFIs by Predefined Antibody Groups in the Proficiency Panel

**FIGURE 3:**
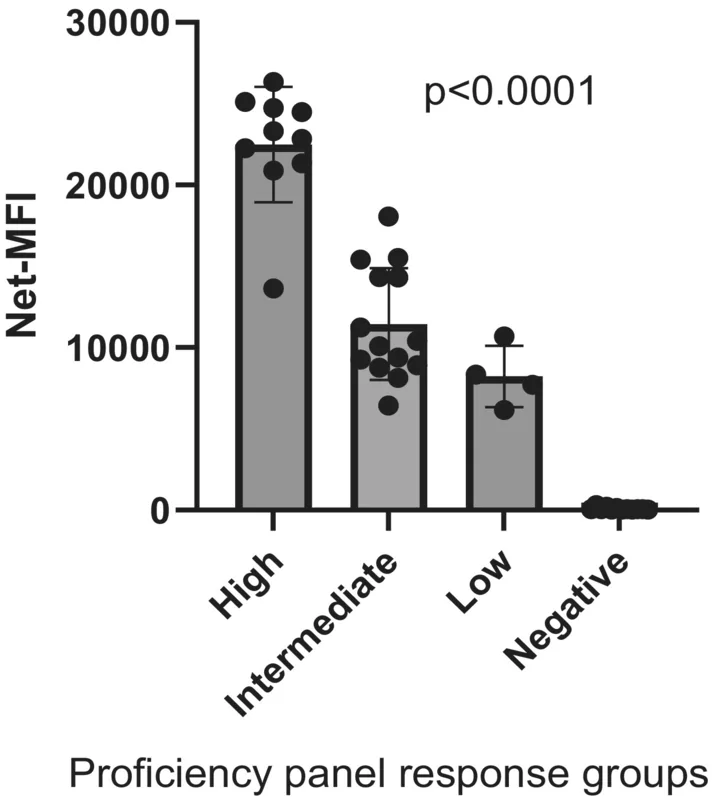
HPV‐11 IgG Net‐MFI distributions within predefined antibody response groups. The bar graph represents the median Net‐MFI IgG values against HPV‐11 for proficiency samples predefined as high, intermediate, low, and negative response groups. Individual data points are shown for each sample within the respective groups. Error bars indicate the standard error of the mean (SEM). The median MFIs for HPV‐11 IgG were significantly different by the predefined antibody groups (*p* < 0.0001).

The median MFIs for all IgGs reported by our L1‐Luminex assay were significantly different across the predefined antibody groups in the proficiency panel, confirming the assay's ability to differentiate between high, intermediate, low, and negative response groups (all *p* < 0.0001). Representative results are presented for HPV‐11 IgG, illustrating median MFIs for the high, intermediate, low, and negative response groups of 23,068, 1024, 8024, and 59 (Figure 3). For HPV‐6, median IgG MFIs were 17,620, 11,469, 5690, and 68, respectively. For HPV‐16, median IgG MFIs were 9248, 5720, 2636, and 34, respectively. For HPV‐18, median IgG MFIs were 12,913, 3320, 2231, and 43, respectively. For HPV‐35, median IgG MFIs were 1531, 475, 171, and 39, respectively. For HPV‐45, median IgG MFIs were 1143, 885, 300, and 12, respectively. For HPV‐58, median IgG MFIs were 11,022, 6382, 4396, and 23, respectively.

### 3.8 Distribution of IgA MFIs by HPV and Overall Correlation With IgG

IgA antibody levels had a Median MFI of 46 for HPV‐6, 193 for HPV‐11, 56 for HPV‐16, 28 for HPV‐18, 12 for HPV‐35, 34 for HPV‐45, and 73 for HPV‐58 (Figure 2, top right panel). Among the high response group, the net MFI values for IgA across all strains of HPV‐6, 11, 16, 18, 45, and 58 ranged between 3 and 6312 (Figure 2, top right panel). Net‐MFI for IgA in the intermediate response group ranged from 4 to 1946 (Figure 2, top right panel). IgA was highly correlated with IgG (Spearman's *ρ* = 0.74, *p* < 0.0001, Figure S1).

## 4 Discussion

Based on the results of this study, the L1 polyclonal binding assay may be a potential alternative to the existing serologic assays but needs further validation. The L1 protein, the major capsid protein of HPV, was thought to be inferior to the VLP antigen because of earlier studies suggesting the need for an antigen with secondary structure [13, 32, 33]. However, our data suggest that the L1 antigen alone was able to differentiate a set of blinded samples with varying levels of IgG antibody, with sensitivity and specificity reaching 100% for most HPV types. Moreover, HPV‐45 VLP has historically had a weaker antibody response in ELISA assays with sensitivity as low as 68.4% and specificity as low as 60.9%, suggesting a suboptimal antigen [31]. In contrast, our assay had a 100% and 92.9% sensitivity at +3 SD and +5 SD, respectively, suggesting we may have identified an alternative HPV45 antigen for antibody assays.

VLP‐based assays are the gold standard for HPV serology because they capture type‐specific antibody responses that recognize the conformational epitopes of HPV [12, 17, 34, 35]. While these assays are highly sensitive and specific, the production of individual VLPs for each HPV genotype is labor‐intensive and resource demanding. VLP generation requires the expression and assembly of multiple recombinant viral proteins in specialized cell lines [32, 36]. Multiplexing the assay for several different genotypes of HPV adds further complication and is only available in certain research laboratories [20, 22, 23]. Moreover, the process is time‐consuming and requires careful quality control to ensure the correct assembly of VLPs [37]. With the expansion of vaccination programs worldwide, there is a need for assays that are scalable and easy to implement for studies of population level immunity.

The glutathione S‐transferase (GST)‐L1 multiplex serology assay was favored for use in sero‐epidemiologic studies because, unlike the VLP‐based assays, it reduced the complexity and cost of production but also enhanced assay throughput, making it suitable for population studies [26, 27]. When the GST‐L1 assay was compared to other VLP‐based assays, it had a lower sensitivity for immunity, either from natural infection or a single dose of vaccination, leading to misclassification of antibody positive as seronegative [27, 28, 38]. Although sample sizes, non‐specific background, and seropositivity cutoffs may have contributed to the assay's lower sensitivity, the GST‐L1 assay may not be the best candidate for monitoring populations with variable antibody responses.

One notable finding in our study was seroreactivity to HPV‐35, a genotype not included in the 9‐valent HPV vaccine. Prior non‐human studies have observed some HPV‐35 seroreactivity [39, 40, 41]. However, the detection observed in one animal study may be because the investigators used an L1/L2 chimeric virus particle that included an L2 with ≥ 90% peptide similarity to multiple oncogenic HPVs, including HPV‐35. The detection we observed could also be because of cross‐reactivity with HPV16 antibodies generated from the vaccine. Another animal study [40] found that antibody HPV16.I23 after immunization with HPV L1 VLPs cross‐reacted with a linear epitope present on HPV‐35. Interestingly, another study found that HPV‐35 antisera had specificity not just to conformationally dependent epitopes, but also denatured HPV‐35 particles, suggesting HPV‐35 antibodies may bind a linear epitope [41]. Without HPV‐35 included in current vaccines, further population studies are needed to understand HPV‐35's seroreactivity.

In our exploratory analysis, we found the L1‐based polyclonal assay detected IgA in the sample set of proficiency panel samples. The IgG and IgA MFIs correlated with each other, which is consistent with findings from another study [42]. While IgG is thought to be the primary mediator of protection following HPV vaccination [43], the role of IgA remains less well defined. It is currently unknown whether vaccine‐induced IgA directly contributes to protective immunity, though it may indicate mucosal immune activation [44, 45, 46]. This may be particularly relevant for the female reproductive tract, where IgA plays a vital role in local defense [47, 48]. Our data should be interpreted cautiously, as we lacked standardized controls to validate cutoff values. Nonetheless, further development of an IgA assay could lead to new hypotheses about whether the presence of both IgA and IgG provides additional insights into neutralizing or protective antibody profiles.

Despite these promising results, our study has some limitations. Our cut‐offs were determined by using samples from infants and children. While this age group minimizes exposure to HPV, it runs the risk of setting cut‐offs too low because adults may have other exposures that contribute to non‐specific background. Future studies could remedy this limitation by including plasma from a low‐risk adult HPV group that was not available in our study [49]. Another potential shortcoming of our work was that the sample set from the proficiency panel included only 40 samples, which, while informative, is a small sample size. Furthermore, no other assay performance characteristics were evaluated, such as precision, reproducibility, lowest level of detection, and cross‐reactivity. To address this limitation, we plan to continue to validate the assay with additional samples and further testing to address these issues. Further work will include HPV‐type specific positive control standards that have become available to the scientific community from the National Institute for Biological Standards and Controls (NIBSC) [31, 50, 51, 52]. Using these standards will allow us to calibrate our assay to international units for future comparisons across serological assays. Additionally, our assay will need to be compared with standard VLP‐based assays using traditional VLPs as well as neutralization assays to assess its performance.

In conclusion, our L1‐based polyclonal assay offers a potentially scalable, sensitive, and specific method for assessing HPV type‐specific antibody responses. It was able to distinguish varying antibody responses with no false positive rates. After further validation, future studies could assess our assay's ability to detect the durability of antibody responses and investigate its ability to prospectively monitor changes at the population level.

## Author Contributions

Arash Aslanabadi contributed to study design, performed the experiments, analyzed and interpreted the data, and drafted the manuscript. Maryam Karimi, Laura Powell, Lisa Schumaker, Ashley Shutt, Mahsa Hojabri, and Rahim Abbasi contributed to data acquisition, technical support, and critical revision of the manuscript. Søren M. Bentzen contributed to statistical analysis and interpretation of the data. Kevin J. Cullen, Trevor A. Crowell, Rebecca G. Nowak, and Mohammad M. Sajadi contributed to study conception, supervision, interpretation of the results, and critical revision of the manuscript. Rebecca G. Nowak and Mohammad M. Sajadi supervised the project. All authors reviewed and approved the final version of the manuscript.

## Funding

This work was supported by the National Cancer Institute at the National Institutes of Health [1K07CA225403 to R.G.N.] and [R21DE031516 to R.G.N.]; the Maryland Department of Health's Cigarette Restitution Fund Program [CH‐649‐CRF to R.G.N.] and the U.S. Department of Defense through agreements with the Henry M. Jackson Foundation for the Advancement of Military Medicine Inc. [W81XWH‐11‐2‐0174 to T.A.C., W81XWH‐18‐2‐0040 to T.A.C., HT9425‐24‐3‐0004 to T.A.C.]. The authors acknowledge that this work is Patent Pending.

## Conflicts of Interest

The authors declare no conflicts of interest.

## Supporting information


**Figure S1:** Scatter plot of HPV IgG and IgA MFIs with *X* and *Y*‐axis on the logarithmic scale.

## Data Availability

The data that support the findings of this study are available from the corresponding author upon reasonable request.
