# Supplementary material for: Preliminary Development and Assessment of a Multiplex Assay for Human Papillomavirus Antibody Detection Using Commercial L1 Proteins
Source: J Clin Lab Anal. 2026 Sep 1;40(19):e70343. doi: 10.1002/jcla.70343 (PMC13534992; doi:10.1002/jcla.70343)
Supplement: Supplementary file 1 [file JCLA-40-e70343-s001.docx]

**Supplemental Figure 1.** Scatter plot of HPV IgG and IgA MFIs with X and Y-axis on the logarithmic scale.
